# Potential Adverse Effects of Sugammadex Administration: A Scoping Review

**DOI:** 10.7759/cureus.77666

**Published:** 2025-01-19

**Authors:** Wei Ming Liang, Danny Nguyen, Gowtham Anche, Robert Valencia, Luis Periel, Brian Obinero, Mikhail Volokitin

**Affiliations:** 1 Anesthesiology, Touro College of Osteopathic Medicine, New York, USA; 2 Osteopathic Manipulative Medicine, Touro College of Osteopathic Medicine, New York, USA

**Keywords:** adverse effect, anaphylaxis, nmba, reversal, sugammadex

## Abstract

Sugammadex, a novel agent for reversing neuromuscular blockade, has become the preferred method for reversal over the combination of neostigmine and glycopyrrolate due to its lower risk of adverse effects and high efficacy in reversing paralytic agents. Its primary theorized adverse effect is its ability to bind to steroids, which may compromise the effectiveness of hormonal birth control. Further investigation is needed to identify unanticipated or poorly understood side effects and better inform preoperative assessments and use across diverse patient populations.

To gather existing research on sugammadex, with a particular focus on identifying gaps in knowledge regarding its adverse effects and optimizing preoperative evaluation strategies to enhance patient outcomes.

A literature search was conducted by RV, GA, WL, LP, BO, and DN between January and March 2024 using PubMed and Google Scholar. The keywords used were "sugammadex," "adverse effects," and "side effects." Studies published between January 2019 and March 2024 were included, as prior reviews addressed adverse effects reported before 2019. The initial search yielded 1,236 studies, narrowed to 46 after applying eligibility criteria. From these, 20 studies were selected for in-depth analysis, including case reports and case series.

Higher doses of sugammadex have been associated with an in vitro decrease in coagulation factors and a possible increased risk of bleeding. Cases of severe bradycardia, including instances leading to cardiac arrest, have been reported, particularly in pediatric patients and certain adult populations. In pediatric patients, sugammadex was also found to affect heart rate variability following neuromuscular blockade reversal. Anaphylactic reactions were observed at an incidence rate of two in 19,821 patients in a single-center cohort study.

Sugammadex’s early adoption in clinical settings, favored for cases with extensive neuromuscular blockade and significant comorbidities, reflects its clinical value. Adverse effects associated with its use include severe bradycardia, hypotension, a theoretical decrease in the effectiveness of oral contraceptives, and anaphylaxis. Efficacy in the special populations, such as patients who are pregnant is still relatively unexplored, and moving forward, usage of sugammadex should be considered with these adverse effects in mind as more extensive data becomes available.

## Introduction and background

The discovery of sugammadex, a modified gamma-cyclodextrin, in 2007 and its FDA approval in 2015 marked a significant breakthrough in anesthesia. It has rapidly become the most widely used agent for reversing neuromuscular blocking agents (NMBAs), replacing the traditional combination of neostigmine and glycopyrrolate.

NMBAs are classified into two categories: depolarizing and non-depolarizing agents. Depolarizing agents, such as succinylcholine, bind to nicotinic receptors at the neuromuscular junction (NMJ), causing endplate depolarization. Unlike acetylcholine, succinylcholine is not broken down by acetylcholinesterase, so it remains bound to the receptor until the receptor becomes unresponsive, producing a blocking or paralytic effect. In contrast, steroidal non-depolarizing agents like rocuronium, function as competitive antagonists at nicotinic receptors, competing with acetylcholine to inhibit signal transmission.

The reversal of succinylcholine occurs spontaneously as the drug is eventually metabolized by pseudocholinesterase, allowing the receptors at the neuromuscular junction (NMJ) to recover. Using neostigmine for reversal depends on the phase of succinylcholine's effect. Neostigmine enhances Phase I (depolarization of the NMJ) while antagonizing Phase II (desensitization) [1].

Rocuronium and other steroidal non-depolarizing NMBAs are reversed by neostigmine, which increases acetylcholine levels to compete with and displace rocuronium at the nicotinic receptors. However, increased acetylcholine levels can lead to cholinergic side effects such as bradycardia, hypotension, and bronchoconstriction. The co-administration of glycopyrrolate, a muscarinic receptor blocker, mitigates these side effects.

Sugammadex, by contrast, binds to free rocuronium molecules in the plasma, creating a concentration gradient. This gradient pulls rocuronium away from the NMJ and back into the plasma. Since sugammadex does not interact with acetylcholine receptors, it avoids causing cholinergic side effects.

Sugammadex is preferred over neostigmine as a reversal agent due to its superior efficacy, even in cases of deeper blockade, its association with fewer postoperative complications (as demonstrated by the STRONGER study), and its lack of direct interaction with receptors [2].

A review analyzing 4,206 patients across 41 studies comparing sugammadex to neostigmine showed that sugammadex was more effective in both the speed of reversal and its ability to reverse deeper blocks, as measured by the train-of-four ratio [3]. The 2020 STRONGER study, which included 45,172 participants, compared postoperative complications between the two agents and found a lower incidence of complications such as pneumonia and respiratory failure in the sugammadex group [2].

However, sugammadex is not without side effects. Case reports continue to describe complications associated with its use. Literature reviews conducted in 2021 and 2022 analyzed 33 cases from 23 studies and 25 cases from 19 studies, respectively, highlighting similar findings of sugammadex-associated anaphylaxis [4,5]. Reported symptoms included hypotension, erythema, and desaturation. This study aims to analyze additional case reports to gain a deeper understanding of the complications linked to sugammadex.

## Review

Methods

A literature search was conducted by authors WL, DN, BO, LP, GA, and RV between January and March 2024 using the databases PubMed and Google Scholar to identify case reports, case series, retrospective chart reviews, and other studies based on case reports reporting adverse effects associated with sugammadex. The search was performed using the keywords "sugammadex," "adverse effects," and "side effects." The author team decided to focus on studies published between January 2019 and March 2024. Even though sugammadex was approved for use in 2008 by the European Union [6] and in 2015 for the US [7], earlier background research into this topic showed existing literature reviews looking at adverse events from sugammadex administration from 2017 [8]. 

Inclusion criteria were studies that were published between the January 2019 and March 2024 time frame, were one of the study types of interest (case reports, case series, retrospective chart reviews, or observational studies based on case reports), had sugammadex usage in a clinical setting to reverse anesthesia, and published in English. Exclusion criteria were studies with insufficient detail, review studies, studies that failed to identify sugammadex as the inciting agent, preclinical studies, and studies that used other reversal agents in addition to sugammadex. The inclusion and exclusion criteria were agreed upon by authors WL, DN, BO, LP, GA, and RV.

**Figure 1:**
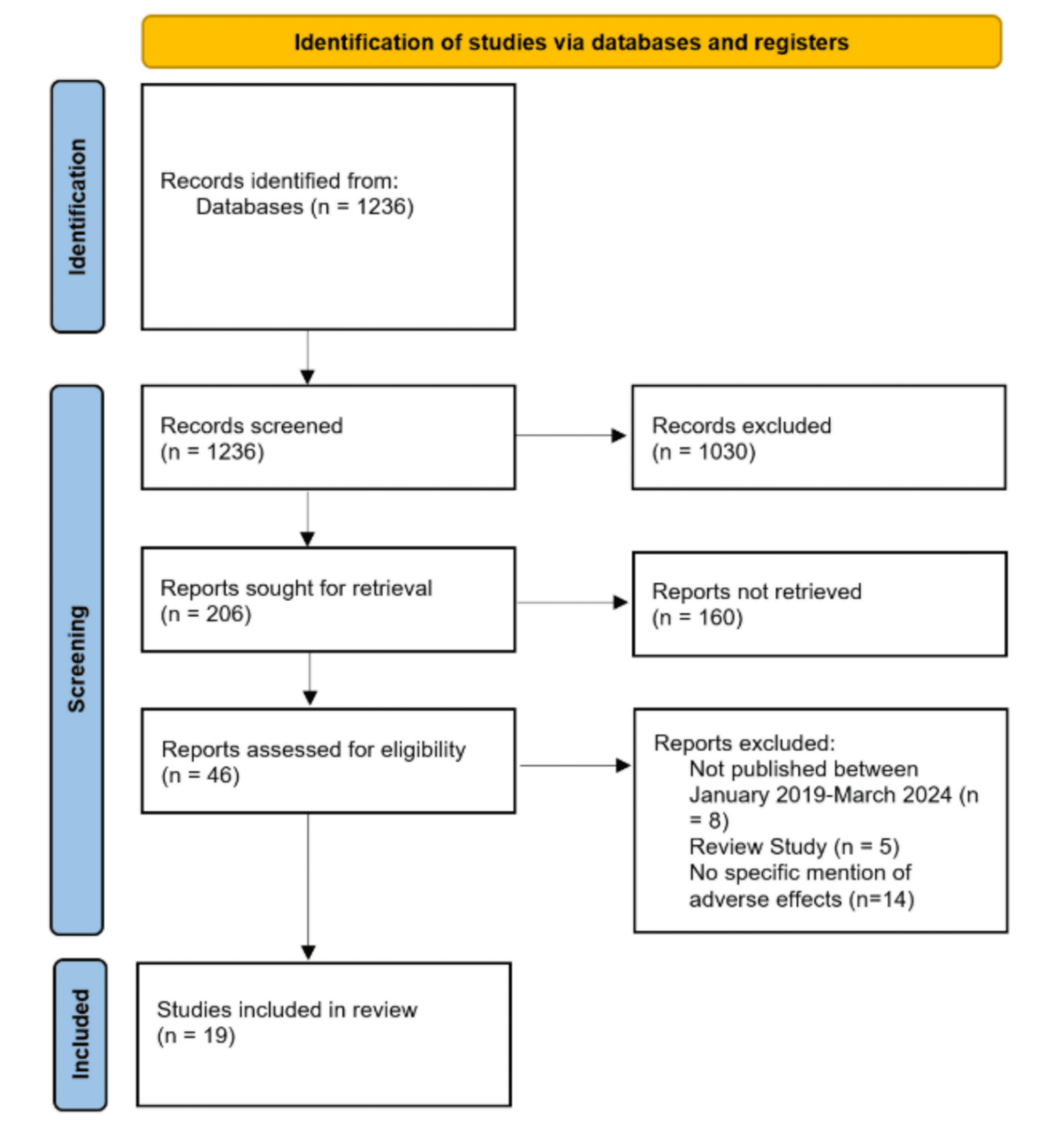
PRISMA diagram for study selection. PRISMA: Preferred Reporting Items for Systematic Reviews and Meta-Analyses.

The initial search using the keywords listed above yielded 1,236 results related to the keywords (Figure 1). Each of the six authors mentioned above was randomly assigned to review 206 results to manually apply the inclusion and exclusion criteria. A second randomly assigned author reviewed the decisions made in the first round and applied the inclusion and exclusion criteria to the 206 results as well. Any discrepancies between these two reviewers were broken by having a third randomly selected author review the result to make a final decision. These two rounds of review refined the pool to 46 studies. These 46 studies underwent a final round of criteria analysis by the entire author pool which narrowed the selection to 19 studies for deeper analysis in the scoping review.

Data from these studies were extracted, including patient demographics (age, gender, comorbidities), sugammadex dosages, and reported adverse reactions. Analysis of the selected articles showed that adverse effects could be categorized into two major groups such as anaphylactoid reactions and bradycardia, with an additional subgroup that encompassed other observed toxicities such as increased bleeding risk and cardiotoxicity. None of these subgroups were established or defined prior to the literature search. Clinical outcomes and interventions were also recorded. Due to the case-based nature of most studies, descriptive statistics were used to summarize the findings, and no hypothesis testing was performed.

Results

A summary of the findings of each study is detailed in Table 1. The most common adverse events found in the selected studies were anaphylactoid reactions and bradycardia. Across seven of the 19 studies analyzed, there were eight instances of anaphylaxis reported. Across eight of the 19 studies analyzed, there were 26 cases of bradycardia reported.There was one instance of prolonged prothrombin time (PT), one report of ST elevations, one occurrence of 2nd-degree Mobitz I heart block, one situation of pulseless electrical activity, and one event of bronchospasm.

**Table 1:** Brief summary of case studies analyzed. PT: prothrombin time.

Author(s)	Year	Type of study	Adverse effect
Choi et al. [9]	2020	Case Report	Anaphylaxis
Burbridge MA [10]	2021	Single-Center Cohort Study	Anaphylaxis
Escher and Cohen [11]	2019	Case Report	Anaphylaxis
Lazo Uslar et al. [12]	2022	Case Reports	Anaphylaxis
Kadiyalaet al. [13]	2023	Case Report	Anaphylaxis
Dichtwald et al. [14]	2023	Case Report	Anaphylaxis
Vaswani et al. [15]	2023	Case Report	Bradycardia, cardiac arrest
Alsuhebani et al. [16]	2020	Prospective Observational Study	Bradycardia
Carvalho et al. [17]	2023	Case Series	Bradycardia
Teng et al. [18]	2021	Case Report	Bradycardia
Yoshida et al. [19]	2020	Case Report	Bradycardia
Fierro et al. [20]	2021	Case Report	Bradycardia, asystole
Pereira et al. [21]	2023	Case Report	Bradycardia, cardiac arrest
Weerasuriya et al. [22]	2024	Case Report	Bradycardia, asystole
Kang et al. [23]	2020	Coagulation Assessment (Thromboelastography)	Prolonged PT, coagulation
Boo et al. [24]	2023	Case Report	Cardiac arrest, coronary vasospasm
Abdelrahim et al. [25]	2023	Case Report	Heart block, spontaneous resolution
Katerenchuk et al. [26]	2024	Case Report	Pulseless electrical activity
Kim et al. [27]	2023	Case Report	Bronchospasm

The first major group of side effects identified was anaphylactoid reactions. Across seven of the 19 studies analyzed, there were eight instances of anaphylaxis reported. One case reported a 60-year-old male who developed hypotension and urticaria after sugammadex reversal requiring ephedrine, phenylephrine, fluids, methylprednisolone, hydrocortisone, and pheniramine [9]. A single-center retrospective chart review of 19,821 patients who received 23,446 doses of sugammadex showed two instances of anaphylaxis [10]. One more case reported a 67-year-old female patient who developed tachycardia, hypotension, and a maculopapular rash following sugammadex administration requiring phenylephrine, fluids, epinephrine, vasopressin, diphenhydramine, and famotidine [11]. One study reported two cases of anaphylaxis: the first was a 62-year-old male patient who developed low cardiac output after sugammadex reversal needing norepinephrine and a nine-year-old girl who developed erythema and hypoxemia requiring intubation, fluids, steroids, and epinephrine [12]. Another case reported was of a 68-year-old woman who developed hypotension, a diffuse erythematous rash, and hypoxemia that progressed into a cardiac arrest [13]. One more report of a 47-year-old male patient mentioned the development of prolonged anaphylactic shock that required continuous, short-term veno-venous hemofiltration [14]. 

The second major group of side effects identified was bradycardia. Across eight of the 19 studies analyzed, there were 26 cases of bradycardia reported (Table 2). One case reported was an eight-month-old girl with complex congenital heart disease who experienced a 10-minute bradycardiac arrest requiring Pediatric Advanced Life Support interventions after sugammadex [15]. A prospective observational study looked at the heart rates of 221 children following sugammadex administration; 18 instances of bradycardia were seen with a correlation to cardiac comorbidities and male sex [16]. Another study reported two instances of bradycardia in pediatric patients that were reversed with atropine [17]. One case report was of an 82-year-old female patient who developed bradycardia after sugammadex that responded to atropine [18]. Supporting this finding, a case reported was a 50-year-old woman who developed bradycardia that did not respond to atropine leading to adrenaline usage [19]. Another case report was of an 80-year-old male patient who developed bradycardia after sugammadex administration that did not respond to atropine and progressed to asystolic cardiac arrest requiring cardiopulmonary resuscitation [20]. Another similar case reported was a 68-year-old male patient who also developed bradycardia refractory to ephedrine after sugammadex that progressed to pulseless electrical activity cardiac arrest [21]. A case of bradycardia progressing to asystole was reported in a 26-year-old female patient who did not respond to atropine requiring two minutes of chest compression for resolution of symptoms [22].

**Table 2:** Summary of bradycardia findings. BPM: beats per minute.

Author	Time to onset of bradycardia	Bradycardia severity
Vaswani et al. [15]	10 minutes	46 BPM
Alsuhebani et al. [16]	2 minutes (median onset)	N/a
Carvalho et al. [17]	Immediately after the administration of sugammadex	53 BPM, 55 BPM
Teng et al. [18]	1 minute	34 BPM
Yoshida et al. [19]	4 minutes	36 BPM
Fierro et al. [20]	1 minute	<35 BPM
Pereira et al. [21]	1 minute	45 BPM
Weerasuriya [22]	1 minute	27 BPM

A study found that sugammadex administration of either 2 or 4 mg prolonged PT. Administration of 4 mg of sugammadex also prolonged the reaction (R) time, even though the value still remained within the normal limits [23]. Another case reported was of a 57-year-old male patient post sugammadex administration who developed ST elevations in the inferior leads after progressing to pulseless electrical activity from right coronary artery (RCA) collapse that responded to intra-coronary nitroglycerin administration [24]. One case reported was of a transient second-degree, Mobitz type I heart block that resolved spontaneously [25]. An additional case was reported of pulseless electrical activity 20 minutes after PACU admission of an 84-year-old male patient who underwent uneventful extubation that was preceded by sugammadex administration [26]. One case reported was a 52-year-old woman who experienced bronchospasm after administration of sugammadex requiring salbutamol [27].

Discussion

This literature review compiled the adverse effects of sugammadex to better understand its safety profile. The most common adverse events found in the selected studies were anaphylactoid reactions and bradycardia. Eight cases of anaphylactoid reactions were found in this search along with 26 reports of bradycardia following administration of sugammadex. There were no identifiable common underlying factors among the cases of anaphylactoid reactions. More cases of bradycardia were seen in pediatric populations but cases were also reported in adult patients. Both toxicity groups had cases that progressed into cardiac arrest. Three of the five cases of adverse effects that did not fall into either category also involve cardiotoxicity in some manner. In total, there were 29 cases of cardiotoxicity across 11 of the 19 studies analyzed. This finding correlates to findings from previous literature reviews where cardiotoxicity was the most prominent adverse event reported [28].

The clinical importance of this literature review expands upon adverse events reported. Cardiotoxicity was the most common adverse event with 29 cases ranging from transient atrioventricular (AV) block to bradycardia corrected by atropine to pulseless electrical activity. Previous studies have shown that the etiology of anaphylaxis is highly variable; previous studies have mentioned biphasic attacks, sugammadex reactive IgE and IgG antibodies, and rocuronium-sugammadex complexes [29]. Patients in both the bradycardia and anaphylactic reactions cases had a broad and diverse medical history highlighting the need for rigorous monitoring during the reversal process and detailed patient history taking before surgical cases. 

While the low chance of adverse events is beneficial to patients, as shown in the STRONGER study, analysis to identify underlying etiology or predictive risk factors can be difficult due to the relative scarcity of reported adverse effects. Another weakness of this study comes from the heavy use of case reports to draw conclusions about sugammadex’s adverse effects. The lack of randomized controlled trials (RCTs) makes it difficult to find evidence if there are any root causes driving reports of adverse events following sugammadex administration. One more possible weakness of this study comes from the geographic distribution of the selected studies; more than half of the studies and case reports come from countries outside the US indicative of sugammadex’s later approval in the US compared to other countries [30]. While there is not enough evidence to support a genetic component driving adverse responses to sugammadex, this skew can obscure the actual incidence, prevalence, and driving factors of adverse events from sugammadex administration.

Future studies should continue to build on prior literature reviews by developing clearer patient profiles. More case reports from the US will be useful in identifying potential adverse effects from sugammadex previously unseen and in determining if there is a geographic component to adverse medication reactions. As sugammadex continues to be used more frequently as a reversal agent, continuous monitoring and analysis of adverse events is necessary to ensure the benefits of this medication are not outweighed by its rare but not insignificant adverse effects [31].

## Conclusions

Sugammadex use for neuromuscular reversal induced by rocuronium or vecuronium is steadily increasing in many institutions across the US. Compared to other agents used for neuromuscular blockade, sugammadex has so far demonstrated a relatively safer drug profile in certain patient populations. Studies have highlighted serious complications including severe anaphylactic reactions and bradycardia. Instances of bradycardia were more common in the pediatric population. Cardiac abnormalities reported include instances of tachycardia, hypotension, or pulseless electrical activity. Other adverse effects have been described such as increased bleeding risk from prolonged coagulation times, or decreased effectiveness of oral contraceptives.

There is a need for RCTs to further objectively illustrate adverse effect profiles, as solely a review of case studies can introduce some unknown factors of biases in different subpopulations. Pertinent populations to study based on the current literature include pregnant patients, pediatric patients, and patients with cardiac comorbidities. Approved by the FDA in 2015, sugammadex suffers from a lack of extensive studies on adverse effects in specific subpopulations, highlighting the need for ongoing clinical monitoring as its use grows.
